# Welcome to *Implementation Science*

**DOI:** 10.1186/1748-5908-1-1

**Published:** 2006-02-22

**Authors:** Martin P Eccles, Brian S Mittman

**Affiliations:** 1 Centre for Health Services Research, University of Newcastle, Newcastle upon Tyne, UK; 2 VA Center for the Study of Healthcare Provider Behavior, VA Greater Los Angeles Healthcare System, Sepulveda, California, USA; 3 Department of Health Services, UCLA School of Public Health, Los Angeles, California, USA

## Abstract

Implementation research is the scientific study of methods to promote the systematic uptake of research findings and other evidence-based practices into routine practice, and, hence, to improve the quality and effectiveness of health services and care. This relatively new field includes the study of influences on healthcare professional and organisational behaviour.

*Implementation Science *will encompass all aspects of research in this field, in clinical, community and policy contexts. This online journal will provide a unique platform for this type of research and will publish a broad range of articles – study protocols, debate, theoretical and conceptual articles, rigorous evaluations of the process of change, and articles on methodology and rigorously developed tools – that will enhance the development and refinement of implementation research. No *one *discipline, research design, or paradigm will be favoured.

*Implementation Science *looks forward to receiving manuscripts that facilitate the continued development of the field, and contribute to healthcare policy and practice.

## Implementation Science

Research continually produces new findings that can contribute to effective and efficient healthcare. However, such research cannot change outcomes unless health services and healthcare professionals adopt the findings into practice. Uneven uptake of research findings – and thus inappropriate care – occurs across settings, specialities and countries [1-3].

Implementation research is the scientific study of methods to promote the systematic uptake of research findings and other evidence-based practices into routine practice, and, hence, to improve the quality and effectiveness of health services. It includes the study of influences on healthcare professional and organisational behaviour.

## Why a new journal?

Recent years have seen the rapid and continuing development of "implementation science", but this has not been accompanied by any dedicated journals. This has hampered the field in three ways. Firstly, implementation research articles are scattered across a wide range of journals, including clinical, public health, health services, and healthcare quality/safety journals. As a result, articles are often difficult to locate, and the breadth of the field is not easily understood. *Implementation Science *will provide a flagship home for this specialized area of research.

Secondly, publication to date has usually been restricted to the final reporting of studies with little or no opportunity to describe the important contextual, developmental and supporting work that would allow for a better interpretation of results and enhance the likelihood of successful replication of an intervention. *Implementation Science *will encompass all aspects of research relevant to the scientific study of methods to promote the uptake of research findings into routine settings in clinical, community and policy contexts. Therefore, in addition to articles reporting final results, *Implementation Science *will publish a broad range of other types of articles, such as: study protocols, debate, theoretical and conceptual articles, rigorous evaluations and reports on the process of change, and articles on methodology and rigorously developed tools. No one discipline, research design, or paradigm will be favoured. The criteria for publication will be relevance to the field, scientific quality, and, as an international journal, generalisability. In pursuit of this end, we are supported by an international, multidisciplinary Editorial Board .

Finally, paper-based journals are bound by the limitations of issue length and frequency. As an electronic journal, *Implementation Science *will be free of such restrictions and will make rapid publication decisions on the basis of the criteria described above. Therefore, articles will be published online immediately upon acceptance (following peer review) and will soon after be listed in PubMed.

## Open access

*Implementation Science *follows the open access policy, changing the way in which articles are published. All articles are freely and universally accessible online, so an author's work can be read by anyone at no cost. The authors hold copyright for their work and may grant anyone the right to reproduce and disseminate the article, provided that it is correctly cited and no errors are introduced [4]. Further, a copy of the full text of each open access article is permanently archived in other online repositories separate from the journal. *Implementation Science *articles are archived in several locations, including: PubMed Central [5], the US National Library of Medicine's full-text repository of life science literature, and in repositories at the University of Potsdam [6] in Germany, at INIST [7] in France, and in e-Depot [8] – the National Library of the Netherlands' digital archive of all electronic publications.

Open access has four broad benefits for science and the general public. First, authors are assured that their work is disseminated to the widest possible audience, given that there are no access barriers. This is accentuated by the authors' right to freely reproduce and distribute their work; for example, by placing it on their institution's website. Second, the information available to researchers will not be limited by their library's budget; the widespread availability of articles will enhance literature searching and use [9]. Third, the results of publicly funded research will be accessible to all, and not just to those with access to a library with a subscription. As such, open access could increase public interest in, and support of, research. The National Institutes of Health (NIH) policy on public access to research calls on researchers to publicly release articles from research supported by NIH in the United States as soon as possible, and within 12 months of final publication [10]. This policy is likely to be replicated by other major funders worldwide. Fourth, a country's economy will not influence its scientists' ability to access articles because resource-poor countries (and institutions) will be able to read the same material as scientists, as well as the general population, of wealthier countries (subject to internet access constraints [11]).

## Journal content and features

*Implementation Science *will offer several features to enhance its value and role in strengthening the field. Timely announcements of new funding opportunities and significant events (e.g., conferences, private or public sector initiatives) will facilitate broad awareness and access to these resources. The publication of conference proceedings and special issues highlighting specific research programs will broaden access to their insights and contributions, for larger audiences.

Several types of planned thematic articles also will strengthen the field. Articles assessing the state of the science, as well as the barriers and challenges researchers face, will facilitate the development of effective solutions, such as new theories, frameworks, and research standards, approaches and methods. Other planned thematic articles will offer practical guidance and insights to implementation researchers. The series labelled "The Practice of Implementation Science" will offer practical, science-based guidance to researchers facing the need to select among available theories, frameworks, research approaches and designs, methods and other options. A planned series of articles written by implementation research users (in the policy and practice communities) will offer implementation researchers insights into the needs, values and preferences of these stakeholders. Enhancing communication between implementation researchers and implementation research users represents an important goal of the journal.

On behalf of the Editorial Board, we hope you will support us in our efforts to make *Implementation Science *a success, through submission of articles, ideas for journal features and content, and involvement in journal activities such as peer reviewing. We look forward to your involvement and suggestions for improving the journal as it develops.

## Competing interests

The author(s) declare that they have no competing interests.
